# Serum and Fecal 3-Bromotyrosine Concentrations in Dogs with Chronic Inflammatory Enteropathy: Clinical Parameters and Histopathological Changes

**DOI:** 10.3390/ani13172804

**Published:** 2023-09-04

**Authors:** Panpicha Sattasathuchana, Naris Thengchaisri, Yasushi Minamoto, Tomomi Minamoto, Jonathan A. Lidbury, Jan S. Suchodolski, Jörg M. Steiner

**Affiliations:** 1 Department of Companion Animal Clinical Sciences, Faculty of Veterinary Medicine, Kasetsart University, Bangkok 10900, Thailand; fvetnrt@ku.ac.th; 2 Gastrointestinal Laboratory, Department of Small Animal Clinical Sciences, School of Veterinary Medicine and Biomedical Sciences, Texas A&M University, College Station, TX 77843, USA; yasushi.minamoto@gmail.com (Y.M.); tbanminamoto@gmail.com (T.M.); jlidbury@cvm.tamu.edu (J.A.L.); jsuchodolski@cvm.tamu.edu (J.S.S.); jsteiner@cvm.tamu.edu (J.M.S.)

**Keywords:** 3-bromotyrosine, chronic inflammatory enteropathy, dogs, diarrhea, histological findings, vomiting

## Abstract

**Simple Summary:**

This study aimed to examine the diagnostic sensitivity of 3-bromotyrosine (3-BrY) and compare serum and fecal 3-BrY concentrations between dogs with chronic inflammatory enteropathy (CIE) that showed pathological changes in biopsy samples of the gastrointestinal (GI) tract. A weak correlation was found between serum C-reactive protein and serum 3-BrY concentrations, but no correlation was observed between the canine chronic enteropathy clinical activity index and serum or fecal 3-BrY concentrations. No significant difference in 3-BrY concentrations was found in biological samples between dogs with CIE who had various GI pathological changes. It is worth noting that dogs with CIE often have increased 3-BrY concentrations in both their serum and fecal samples. However, these 3-BrY elevations may not accurately reflect the severity of GI histological findings.

**Abstract:**

Chronic inflammatory enteropathies (CIEs) in dogs involve the infiltration of gastrointestinal tissue with inflammatory cells. This study aimed to assess the sensitivity of serum and fecal 3-bromotyrosine (3-BrY) concentrations in dogs with CIE. The difference in 3-BrY concentrations in dogs with different gastrointestinal (GI) pathological changes was also assessed. In total, 68 dogs with CIE were enrolled in the study. The median serum 3-BrY concentration was 3.3 µmol/L, while the median 3-day mean and maximum fecal 3-BrY concentrations were 38.9 and 63.2 mmol/g of feces, respectively. The median serum C-reactive protein concentration was 45.0 mg/L. The median 3-day mean and maximum fecal α_1_-proteinase inhibitor concentrations were 6.1 and 9 µg/g of feces, respectively. Increased 3-BrY concentrations were observed in 90.9% of CIE dogs based on serum concentrations, 75.8% based on mean fecal concentrations, and 69.4% based on maximum fecal concentrations. A weak correlation (ρ = 0.31, *p* < 0.0118) was found between serum CRP and serum 3-BrY concentrations. There was no correlation between the canine chronic enteropathy clinical activity index and serum or fecal 3-BrY concentrations (*p* > 0.05). Additionally, no significant difference in serum or fecal 3-BrY concentrations was found among CIE dogs with different GI pathological changes (*p* > 0.05). In conclusion, dogs with CIE have increased 3-BrY concentrations in serum and fecal samples. However, 3-BrY concentrations may not accurately indicate the severity of gastrointestinal inflammation.

## 1. Introduction

Chronic inflammatory enteropathy (CIE) is a complex gastrointestinal (GI) tract disorder characterized by chronic inflammation and is believed to have a multifactorial etiology [1,2]. The clinical manifestations of CIE can be persistent or intermittent and are present for a duration exceeding 3 weeks [1,2,3]. Common clinical signs exhibited by dogs with CIE include anorexia, abdominal pain, diarrhea, vomiting, weight loss, or a combination of these symptoms. The diagnosis of CIE requires the exclusion of other GI diseases, histological evidence of GI inflammation, and clinical improvement with treatment trials [1,3,4,5]. Lymphocytic–plasmacytic gastroenteritis and eosinophilic gastroenteritis are the two most common histological findings in dogs with CIE, whereas other inflammatory cell types such as neutrophils, monocytes, and macrophages are less frequently identified [2,3,6]. Multiple biomarkers for CIE have been developed and evaluated for assessing organ function, disease activity and severity, and disease outcome [1,3,7,8,9,10,11,12,13].

The presence of 3-bromotyrosine (3-BrY) is a sign of eosinophil activation [6,14,15]. This stable product is produced when the highly reactive oxygen species hypobromous acid (HOBr) reacts with tyrosine under physiological conditions [16,17,18]. A method for the measurement of the 3-BrY concentration in biological samples from dogs using electron impact gas chromatography–mass spectrometry (EI-GC/MS) has been established and analytically validated [12,19]. Previous studies have demonstrated that both serum and fecal 3-BrY concentrations were higher in dogs with CIE compared to clinically healthy dogs [10,11,12]. The clinical usefulness of the measurement of serum 3-BrY has been assessed in subtypes of dogs with CIE according to the response to treatment trials [10]. Among dogs with CIE, dogs with steroid-responsive diarrhea had higher serum 3-BrY concentrations compared to dogs with food-responsive diarrhea [10]. The correlation between serum and fecal 3-BrY in canine patients remained unknown. Also, the difference in 3-BrY concentrations between CIE dogs with various pathological changes has not been evaluated.

The objectives of this study were to evaluate the diagnostic sensitivity of serum and fecal 3-bromotyrosine (3-BrY) concentrations in dogs with CIE. Additionally, the study aimed to characterize the relationships between serum and fecal 3-BrY concentrations and examine the correlation between 3-BrY concentrations and C-reactive protein (CRP) concentrations as well as the canine chronic enteropathy clinical activity index (CCECAI) score. Finally, the study aimed to compare 3-BrY concentrations between dogs with CIE that had different pathological changes.

## 2. Materials and Methods

### 2.1. Ethical Approval

All sample collection protocols were reviewed and approved by the Institutional Animal Care and Use Committee (approval number #2012-083), and the study adhered to the ARRIVE (Animal Research: Reporting of In Vivo Experiments) guidelines. All owners signed an informed consent form.

### 2.2. Inclusion Criteria and Sample Collection

This study utilized a prospective cross-sectional design to investigate dogs diagnosed with CIE through histological examination. Dogs from multiple veterinary clinics across the USA, diagnosed with CIE between the years 2012 and 2014, were enrolled in the study. In order to ensure the specificity of CIE, other potential causes of chronic GI disease, including endoparasites, pancreatitis, renal or liver failure, tumors, and hypoadrenocorticism, were systematically ruled out. Microscopic fresh fecal examination was performed to exclude GI parasitic infestation, while serum chemistry analysis and measurements of serum concentrations of baseline cortisol and canine pancreatic lipase immunoreactivity (as measured by Spec cPL, Idexx Laboratories, Westbrook, Maine) were performed to exclude systemic diseases, such as renal or liver failure, hypoadrenocorticism, and pancreatitis. Additionally, a questionnaire was completed by all dog owners for the calculation of the canine chronic enteropathy clinical activity index (CCECAI) at the time of enrollment (supplementary data) [1]. The primary care veterinarians assessed the CCECAI scores at the time of collecting EDTA blood and serum samples. Approximately one gram of fresh feces was collected over three consecutive days at home by the dog’s owner, with each sample being placed in an individual fecal container. The collected fecal samples were immediately frozen by the dog’s owner. All biological samples were shipped on ice to the GI Laboratory at Texas A&M University and immediately stored at −80 °C prior to analysis. Gastrointestinal biopsies were collected via endoscopic or laparoscopic procedures by local veterinarians and stored in 10% formalin before being shipped to the GI Laboratory. The modality of biopsy collection (i.e., endoscopic or surgical) was left to the discretion of the primary care veterinarian. The CCECAI score was determined, and blood and fecal samples were collected either less than two weeks before or on the same day as the surgical or endoscopic biopsies were collected. The dogs enrolled had no specific dietary restrictions. Dogs were also not allowed to receive glucocorticoid treatment before sample collection.

### 2.3. Measurement of Serum and Fecal 3-BrY Concentrations

The concentrations of 3-BrY in serum and fecal samples were measured using stable isotope dilution and electron ionization gas chromatography/mass spectrometry, with synthetic deuterated-BrY (D3-bromotyrosine) as the internal standard, as previously described [11,12,19]. A reference interval for canine serum 3-BrY concentration was previously established as ≤0.6 µmol/L based on samples from 41 healthy dogs [11,12,19]. Reference intervals for the 3-day mean fecal 3-BrY and 3-day maximum fecal 3-BrY concentrations established based on samples from 40 healthy dogs were 3.7–23.0 and 3.7–37.8 mmol/g of feces, respectively [12]. Fecal concentrations were based on fecal wet weight and were expressed as mmol/g of feces [12,20,21].

### 2.4. Measurement of Serum C-Reactive Protein Concentrations

A commercially available solid-phase immunoassay was used to measure serum CRP concentrations (Tri-Delta Phase CRP, Tri-Delta Diagnostic, Boonton Township, New Jersey). The reported reference interval for serum CRP concentration was 0–9.9 mg/L [22,23], with a cut point ≥ 10 mg/L for significant inflammatory disease [22,23].

### 2.5. Measurement of Fecal α_1_-Proteinase Inhibitor Concentrations 

Fecal α_1_-proteinase inhibitor (α_1_PI) concentrations were measured in 3-day fecal samples using an in-house radioimmunoassay. The reference interval for fecal α_1_PI concentration was 2.2–18.7 µg/g of feces [20]. The diagnostic cut point for excessive GI protein loss has previously been determined as a mean or maximum fecal α_1_PI ≥ 13.9 or ≥21 µg/g of feces, respectively [20].

### 2.6. Evaluation of Canine Chronic Enteropathy Clinical Activity Index (CCECAI)

The CCECAI is a summative score of nine clinical variables, including the animal’s attitude, appetite, vomiting, fecal consistency, fecal frequency, weight loss, serum albumin concentration, ascites and peripheral edema, and pruritus [1]. Each variable is assessed a score from 0 to 3, and the CCECAI score is the sum of the component scores. A score of 0 is considered normal, 1 is mildly abnormal, 2 is moderately abnormal, and 3 is severely abnormal. A total CCECAI score of less than 4 is considered clinically insignificant disease, while scores ranging from 4 to 5 indicate mild clinical disease, scores from 6 to 8 indicate moderate clinical disease, scores from 9 to 11 indicate severe clinical disease, and scores greater than 11 indicate very severe clinical disease.

### 2.7. Evaluation of GI Histological Tissues 

All GI biopsy tissues were processed and analyzed through the GI Laboratory at Texas A&M University. The evaluation of histopathological findings was performed by board-certified pathologists using the grading system described by the World Small Animal Veterinary Association [2]. Both morphological and inflammatory criteria were evaluated. The morphological criteria included surface epithelial damage, increased gastric lymphoid tissue, damage to the gastric pit epithelium, fibrosis/atrophy of the mucosal layer, shortened villi, distorted or distended crypts, and enlarged lacteals. The inflammatory criteria included the presence of intraepithelial lymphocytes and the infiltration with various inflammatory cells, such as lymphocytes, plasma cells, eosinophils, neutrophils, and macrophages into the lamina propria. The presence or absence of each morphological and inflammatory criterion was considered for statistical analyses. 

### 2.8. Statistical Analyses

All statistical analyses were performed by commercially available software packages (JMP Pro 10, SAS Institute, Cary, NC; PRISM v.6.0, Graph-Pad Software, La Jolla, CA, USA; and STATA version 14.2 StataCorp LLC, College Station, TX, USA). A minimum sample size of 38 subjects was necessary to obtain a statistical power of 0.8 with a significance level of 0.05 when assessing the sensitivity of serum 3-BrY or fecal 3-BrY concentrations for diagnosing CIE in the same subjects.

All data were tested for normality using a Shapiro–Wilk test, and the results were presented as median (range). Mann–Whitney U tests were used to compare 3-BrY concentrations (serum, mean fecal, and maximum fecal) between male and female dogs as well as between purebred and mixed-breed dogs. The diagnostic sensitivity of each marker for detecting CIE was achieved by dividing the number of dogs with CIE with concentrations above the reference interval or cut point by the total number of dogs with CIE and then multiplying the result by 100. A binomial distribution was used to calculate the 95% confidence interval (CI). Spearman’s rank sum correlation test was used to evaluate the correlations among serum 3-BrY, 3-day mean fecal 3-BrY, 3-day maximum fecal 3-BrY, serum CRP, and CCECAI scores. Negligible, weak, moderate, strong, and very strong relationships corresponded to Spearman correlation coefficients of 0.00–0.09, 0.10–0.39, 0.40–0.69, 0.70–0.89, and 0.90–1.00, respectively [24].

## 3. Results

The study included 68 dogs diagnosed with CIE. The dogs had a median age of 6 (0.5–13) years, with 40 males and 28 females (Table 1). Among the 68 dogs, 57 (83.8%) were purebred, with Golden Retrievers being the most prevalent breed (11.8%), followed by Labrador Retrievers (7.4%), Boxers (3; 4.4%), Miniature Schnauzers (3; 4.4%), Australian Shepherds (2; 2.9%), Cavalier King Charles Spaniels (2; 2.9%), Pomeranians (2; 2.9%), Rat Terriers (2; 2.9%), Siberian Huskies (2; 2.9%), Yorkshire Terriers (2; 2.9%), and one dog (1.5%) of each of the following breeds: Akita, American Bull Dog, Basenji, Bernese Mountain Dog, Bichon Frise, Boston Terrier, Australian Cattle Dog, Chesapeake Bay Retriever, English Pointer, Great Dane, Greyhound, Irish Setter, Italian Greyhound, Jack Russel, Maltese, Miniature Poodle, Pekingese, Pitbull, Portuguese Water Dog, Pug, Rottweiler, Soft-coated Wheaten Terrier, Vizsla, Weimaraner, Welsh Terrier, and West Highland White Terrier. Mixed-breed dogs accounted for 16.2% (*n* = 11) of the cohort. 

The median serum 3-BrY concentration was 3.3 (0.6–43.7) µmol/L. The median 3-day mean fecal 3-BrY concentration was 38.9 (3.7–142.2) mmol/g of feces, and the median 3-day maximum fecal 3-BrY concentration was 63.2 (3.7–197.9) mmol/g of feces. The median serum CRP concentration was 5.0 (0–60.0) mg/L. The median CCECAI score was 6 (0–19). Finally, the median 3-day mean fecal α_1_PI concentration and the 3-day maximum fecal α_1_PI concentration were 6.07 (2.2–217.4) and 9 (2.2–227.3) µg/g of feces, respectively. 

There were no significant differences in serum 3-BrY (*p* = 0.5085), 3-day mean fecal 3-BrY (*p* = 0.8896), or 3-day maximum fecal 3-BrY (*p* = 0.9008) concentrations between male and female dogs. Also, there were no significant differences in serum 3-BrY (*p* = 0.3207), 3-day mean fecal 3-BrY (*p* = 0.1339), or 3-day maximum fecal 3-BrY (*p* = 0.2495) concentrations between purebred and mixed-breed dogs.

**Figure 1:**
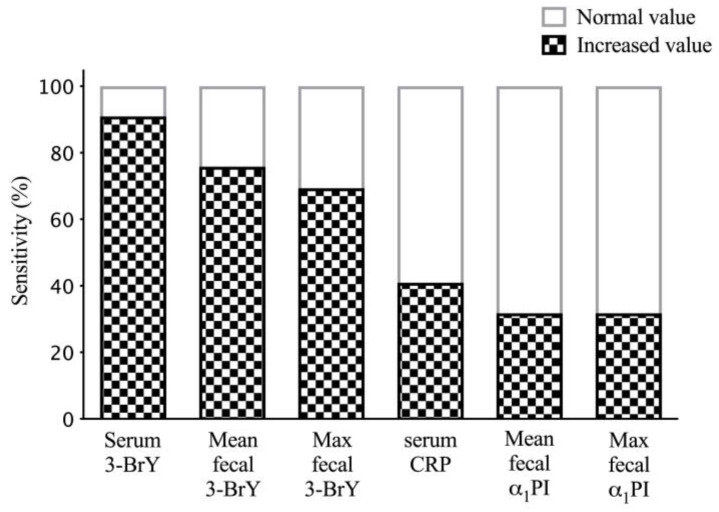
Histogram illustrating sensitivity (%) or number of dogs with chronic inflammatory enteropathy having increased serum 3-bromotyrosine (3-BrY), mean fecal 3-BrY, maximum fecal 3-BrY, serum C-reactive protein (CRP), mean fecal α_1_-proteinase inhibitor (α_1_PI), and maximum fecal α_1_PI concentrations.

Utilizing established reference intervals for serum 3-BrY, 3-day mean fecal 3-BrY, and 3-day maximum fecal 3-BrY concentrations [11,12,19], the diagnostic sensitivities for CIE in dogs were determined. The results demonstrated that 90.9% (95% confidence interval (CI): 81.3–96.6%) of dogs exhibited serum 3-BrY concentrations exceeding the upper limit of the reference interval. Moreover, 75.8% (95% CI: 63.3–85.8%) of dogs displayed 3-day mean fecal 3-BrY concentrations above the upper limit, while 69.4% (95% CI: 56.4–80.4%) of dogs had 3-day maximum fecal 3-BrY concentrations above the upper limit of the reference interval (Figure 1). In contrast, the diagnostic sensitivity was only 40.9% (95% CI: 29.0–53.7%) for serum C-reactive protein (CRP) concentrations and 31.8% (95% CI: 20.6–44.7%) for 3-day mean and maximum fecal α_1_PI concentrations.

A Spearman’s rank correlation test showed a very strong correlation between mean fecal 3-BrY and maximum fecal 3-BrY concentrations (ρ = 0.97; *p* < 0.0001; Table 2). There was a weak correlation between serum CRP and serum 3-BrY concentrations (ρ = 0.31; *p* = 0.0118). There was a moderate correlation between serum CRP concentrations and the CCECAI score (ρ = 0.45; *p* = 0.0002; Table 2). 

The GI biopsy samples were obtained using either endoscopy (gastroduodenoscopy and/or colonoscopy) or surgical methods. Out of the total of 68 dogs with CIE, 49 dogs underwent endoscopic biopsy collection, while full-thickness surgical biopsies were collected for the remaining 19 dogs. Of the collected tissue samples, 59 were collected from the stomach, 60 from the duodenum, 19 from the ileum, and 19 from the colon. There was no correlation between 3-BrY concentrations in biological samples and CCECAI scores (*p* ≥ 0.05; Table 2). There were no significant differences in serum, 3-day mean fecal, or 3-day maximum fecal 3-BrY concentrations between dogs with CIE that had different GI pathological changes (*p* ≥ 0.05; Table 3, Table 4 and Table 5).

## 4. Discussion

Our results revealed that of the markers studied here, serum 3-BrY concentrations had the highest diagnostic sensitivity for CIE in dogs (90.9%). Also, we found a very strong correlation between the 3-day mean and the 3-day maximum fecal 3-BrY concentrations, a weak correlation between serum 3-BrY and CRP concentrations, and a moderate correlation between serum CRP concentrations and CCECAI scores in dogs with CIE. However, there was no correlation between serum or fecal 3-BrY concentrations and CCECAI, nor were associations between serum 3-BrY concentrations and GI histological lesions found.

3-BrY is a distinct byproduct generated from the activation of eosinophil peroxidase [6,15,16,17,18]. After an eosinophil’s respiratory burst, superoxide interacts with eosinophil peroxidase, resulting in the generation of hypobromous acid [15,16,17,18]. Hypobromous acid is a highly reactive oxygen species and serves as an oxidizing agent with antimicrobial properties [15,16,17,18]. When hypobromous acid reacts with tyrosine under physiological conditions, it forms the stable compound known as 3-BrY [6,15,16,17,18]. This study focused on evaluating 3-BrY concentrations in serum and feces to assess the clinical usefulness of measuring 3-BrY concentrations in dogs with chronic enteropathy. Several parameters, including the canine chronic enteropathy clinical activity index (CCECAI) and some established biomarkers, such as CRP and α_1_-PI, were determined in all dogs enrolled in this study to better characterize the dogs enrolled as to the severity of their intestinal inflammatory disease as well as their intestinal protein loss [7,25]. α_1_PI is an acute-phase protein, and its concentrations are used clinically for evaluating dogs for excessive gastrointestinal protein loss [20]. Dogs with CIE may have an impairment of the integrity of the intestinal lining or dilatation of intestinal lacteals, potentially leading to protein-losing enteropathy [20]. The CCECAI has been described as an objective marker of disease severity for dogs with CIE. It is easily obtainable through a questionnaire completed by the owner. It has previously been reported that a higher CCECAI score is associated with a decreased quality of life for the affected dog [1,26].

In the current study, sensitivities for diagnosing CIE in dogs were determined by employing established reference intervals. The findings characterized a notable proportion of dogs exhibiting elevated 3-BrY concentrations (90.9%), whereas the diagnostic sensitivities were considerably lower for serum C-reactive protein (CRP) concentrations (40.9%) and 3-day mean/maximum fecal α_1_PI concentrations (31.8%). The clinical usefulness of serum CRP and fecal α_1_PI concentrations for diagnosing canine CIE may be limited as they have a sensitivity lower than 50%. Hence, the measurement of 3-BrY may prove to be a valuable marker for verifying the diagnosis of CIE in dogs.

The correlations among serum 3-BrY, fecal 3-BrY, serum CRP, and CCECAI were also evaluated (Table 3). Notably, there was a strong correlation between the 3-day mean and maximum fecal 3-BrY concentrations, which suggests that they are closely related. This would be expected as the maximum fecal 3-BrY concentration has a direct impact on the mean fecal 3-BrY concentration [12]. However, determination of both the mean and maximum fecal 3-BrY concentrations requires measurement of fecal 3-BrY in three consecutive fecal samples. Our previous study found that there was a large variation in 3-BrY concentrations for each fecal sample [12]. Therefore, 3-day mean and maximum fecal 3-BrY concentrations should be used to reduce such variability. There was only a weak correlation between serum 3-BrY and serum CRP concentrations in this cohort of dogs. This finding may be due to the fact that while both analytes are increased in response to inflammation, they may be affected differently by different types of inflammatory responses [1]. Therefore, multiple diagnostic tests are necessary to characterize each canine patient with CIE.

Previous studies have evaluated the association between serum CRP concentration and CCECAI scores with conflicting results [1,27]. However, the present study identified a moderate association between serum CRP concentrations and CCECAI scores, suggesting that serum CRP concentrations may be clinically useful as a potential objective marker for assessing disease activity in dogs with CIE. The conflicting results with previous studies [1] can be attributed to variations in animal groups and experimental settings. It should be noted that the present study did not find an association between the CCECAI and serum or fecal 3-BrY concentrations in dogs with CIE [10]. These results suggest that neither serum nor fecal 3-BrY concentrations are useful for evaluating the clinical severity of CIE. Based on the present study, it can be concluded that the presence or absence of various GI pathological changes in dogs with CIE does not significantly affect the serum, 3-day mean, and 3-day maximum fecal 3-BrY concentrations. Therefore, the measurement of 3-BrY concentrations in serum and feces may not indicate the presence and severity of GI histological findings of dogs with CIE.

One limitation of the present study is the inability to determine the diagnostic specificity of the elevated serum and fecal 3-BrY concentrations, as the study did not include control dogs exhibiting GI symptoms caused by other non-gastrointestinal diseases. In addition, this study also did not include any healthy control dogs. However, the WSAVA GI histological grading system was utilized to characterize abnormalities of GI tissues in all dogs with CIE included in this study. Furthermore, our previous studies have demonstrated that dogs with CIE had increased serum and fecal 3-BrY concentrations in comparison to healthy control dogs [10,11,12]. Another limitation is the limited availability of biopsy results from each segment of the GI tract. Inflammation of the GI tract can either be diffuse throughout the entire GI tract or can be localized to one segment or even area [2,28,29,30]. Also, relying on histopathological findings from a single location within the GI tract may not accurately reflect the overall level of inflammation present throughout the GI tract [2,28,29,30]. Additionally, various types of inflammatory cells, such as neutrophils, lymphocytes, eosinophils, and macrophages, can be observed in dogs with CIE [2]. It may be beneficial to employ a combination of multiple biomarkers to assess the activation of inflammatory cells and gain a more comprehensive understanding of the severity of intestinal inflammation in dogs with CIE. 

## 5. Conclusions

The present study revealed that serum 3-BrY concentrations were increased above the upper limit of the reference interval in more than 90% of dogs with CIE. Mean and maximum fecal 3-BrY concentrations were increased above the upper limit of the reference interval in 75% and 69% of dogs with CIE. However, serum and fecal 3-BrY concentrations may not reflect the severity of clinical signs and may not serve as reliable biomarkers for assessing histological severity in dogs with CIE. Further research is needed to better understand the clinical usefulness of 3-BrY in dogs with CIE.

## Figures and Tables

**Table 1:** Characteristics of dog with chronic inflammatory enteropathy (CIE, *n* = 68).

Parameter	Dogs with CIE
Age (years, [range])	6 [0.5–13]
Sex (*n*)	
Male	40 (58.8%)
Female	28 (41.2%)
Body size (*n*)	
Small	19 (27.9%)
Medium	10 (14.7%)
Large	39 (57.4%)

**Table 2:** Spearman correlation coefficients evaluating the relationships of 3-bromotyrosine (3-BrY) with various biochemical parameters, including serum 3-BrY concentration, mean fecal 3-BrY concentration, maximum fecal 3-BrY concentration, and C-reactive protein concentration.

	Mean Fecal 3-BrY Concentration	Maximum Fecal 3-BrY Concentration	C-Reactive Protein Concentration	Canine Chronic Enteropathy Clinical Activity Index
Serum 3-BrY concentration	0.1608	0.2072	0.3129 *	0.1206
Mean fecal 3-BrY concentration		0.9727 **	0.1136	0.1594
Maximum fecal 3-BrY concentration			0.1176	0.1284
C-reactive protein				0.4479 **

* p < 0.05, ** p < 0.01.

**Table 3:** Comparison of serum 3-bromotyrosine concentrations between dogs with absence or presence of various histological findings.

Lesion	Total Number of Dogs	Absence of Lesion			Presence of Lesion		
		N	Median (µmol/L)	Range (µmol/L)	N	Median (µmol/L)	Range (µmol/L)
***Morphologic criteria***							
Surface epithelial injury	48	29	2.8	0.6–15.7	19	4.1	0.6–43.7
Gastric lymphoid hyperplasia	44	32	3.7	0.6–43.7	12	4.0	0.62–12.7
Gastric pit epithelial injury	44	38	3.0	0.6–16.5	6	5.2	2.8–8.9
Mucosal fibrosis/mucosal atrophy	44	33	4.0	0.6–15.9	11	2.1	0.6–43.7
Villous stunting	45	26	3.8	0.6–15.9	19	3.9	0.6–43.7
Crypt distension/distortion	45	21	2.6	0.6–12.7	24	4.0	0.6–43.7
Lacteal dilatation	45	20	2.7	0.6–15.9	25	4.0	0.6–43.7
***Inflammatory criteria***							
Intraepithelial lymphocytes	48	24	3.7	0.8–15.9	24	3.3	0.6–43.7
Lamina propria lymphocytes and plasma cells	48	4	4.9	0.8–6.4	44	3.5	0.6–43.7
Lamina propria eosinophils	48	32	2.7	0.6–43.7	16	4.0	0.6–12.7
Lamina propria neutrophils	48	41	3.6	0.6–43.7	7	4.1	2.4–15.9
Lamina propria macrophages	48	38	3.2	0.6–15.9	10	4.8	0.6–43.7

No statistical significance was found.

**Table 4:** Comparison of 3-day mean fecal 3-bromotyrosine concentrations between dogs with absence or presence of various histological findings.

Lesion	Total Number of Dogs	Absence of Lesion			Presence of Lesion		
		N	Median (mmol/g)	Range (mmol/g)	N	Median (mmol/g)	Range (mmol/g)
***Morphologic criteria***							
Surface epithelial injury	44	28	51.9	3.7–139.0	16	30.2	3.7–127.3
Gastric lymphoid hyperplasia	40	32	40.6	3.7–139.0	8	48.6	3.7–129.2
Gastric pit epithelial injury	40	35	37.6	3.7–131.9	5	111.1	30.0–139.0
Mucosal fibrosis/mucosal atrophy	39	28	40.6	6.7–131.9	11	23.1	3.7–139.0
Villous stunting	41	24	45.0	3.7–131.9	17	33.5	13.1–139.0
Crypt distension/distortion	41	19	30.5	3.7–131.9	22	61.6	3.7–139.0
Lacteal dilatation	41	20	30.2	3.7–111.1	21	63.2	3.7–139.0
***Inflammatory criteria***							
Intraepithelial lymphocytes	44	24	33.8	3.7–139.0	20	67.0	3.7–131.9
Lamina propria lymphocytes and plasma cells	44	4	35.6	6.7–139.0	40	42.2	3.7–131.9
Lamina propria eosinophils	44	32	59.1	3.7–139.0	12	29.6	13.1–107.8
Lamina propria neutrophils	44	36	40.6	3.7–139.0	8	33.6	13.1–107.8
Lamina propria macrophages	44	34	40.6	3.7–139.0	10	38.1	3.7–129.2

No statistical significance was found.

**Table 5:** Comparison of 3-day maximum fecal 3-bromotyrosine concentrations between dogs with absence or presence of various histological findings.

Lesion	Total Number of Dogs	Absence of Lesion			Presence of Lesion		
		N	Median (mmol/g)	Range (mmol/g)	N	Median (mmol/g)	Range (mmol/g)
***Morphologic criteria***							
Surface epithelial injury	44	28	85.4	3.7–167.3	16	44.8	3.7–182.7
Gastric lymphoid hyperplasia	40	32	52.8	3.7–182.7	8	64.8	3.7–141.4
Gastric pit epithelial injury	40	35	47.9	3.7–141.4	5	162.0	54.7–182.7
Mucosal fibrosis/mucosal atrophy	39	28	58.6	12.5–182.7	11	50.8	3.7–173.5
Villous stunting	41	24	83.3	3.7–167.3	17	50.8	24.0–182.7
Crypt distension/distortion	41	19	39.0	3.7–167.3	22	92.0	3.7–182.7
Lacteal dilatation	41	20	46.2	3.7–167.3	21	95.1	3.7–182.7
***Inflammatory criteria***							
Intraepithelial lymphocytes	44	24	46.6	3.7–162.0	20	92.9	3.7–182.7
Lamina propria lymphocytes and plasma cells	44	4	51.3	12.5–162.0	40	65.2	3.7–182.7
Lamina propria eosinophils	44	32	88.5	3.7–173.5	12	38.6	20.7–182.7
Lamina propria neutrophils	44	36	58.6	3.7–173.5	8	56.1	21.5–182.7
Lamina propria macrophages	44	34	58.6	3.7–167.3	10	70.7	3.7–182.7

No statistical significance was found.

## Data Availability

The data presented in this study are available in supplementary data (owner questionnaire).

## Supplementary Materials

The following supporting information can be downloaded at: https://www.mdpi.com/article/10.3390/ani13172804/s1, Supplementary data_CCECAI.
