# Supplementary material for: Serum and Fecal 3-Bromotyrosine Concentrations in Dogs with Chronic Inflammatory Enteropathy: Clinical Parameters and Histopathological Changes
Source: Animals (Basel). 2023 Sep 4;13(17):2804. doi: 10.3390/ani13172804 (PMC10486566; doi:10.3390/ani13172804)
Supplement: Supplementary file 1 [file animals-13-02804-s001.zip › animals-2539993-supplementary.pdf]

☐1st ☐ 2nd ☐ 3rd
Time Submission

TAMU GI Lab – Canine Chronic Enteropathy Clinical Activity Index

Date of evaluation:_________________

Name of dog:____

Breed:_____________________________________ Weight (lbs.):__________________

Age/DOB:___________________________ Sex (circle): male neutered female spayed

Name of owner: ____

Clinic/Hospital:____

Dog evaluated by:____

**Evaluation of:**	**score (circle)**		**Specify, if applicable**
**Attitude/activity**	0	normal	
	1	slightly decreased	
	2	moderately decreased	
	3	severely decreased	
**Appetite**	0	normal	
	1	slightly decreased	
	2	moderately decreased	
	3	severely decreased	
**Vomiting**	0	normal	
	1	mild (1x per week)	
	2	moderate (2-3x per week)	
	3	severe (>3x per week)	
**Stool consistency**	0	normal	
	1	slightly soft feces, or fecal blood, mucus, or both	(please circle)
	2	very soft feces	
	3	watery diarrhea	
**Stool frequency**	0	normal	
	1	slightly increased (2-3x per day)	
	2	moderately increased (4-5x per day)	
	3	severely increased (>5x per day)	
**Weight loss**	0	none	
	1	mild (<5%)	loss:______lbs/kg
	2	moderate (5-10%)	
	3	severe (>10%)	
**Albumin concentration**	0	Albumin > 2.0 g/dL	
	1	Albumin 1.5-1.99 g/dL	
	2	Albumin 1.2-1.49 g/dL	
	3	Albumin <1.2 g/dL	

**Ascites and peripheral edema**	0	none	
	1	mild ascites or peripheral edema	
	2	moderate amount of ascites/peripheral edema	
	3	severe ascites/pleural effusion and peripheral edema	
**Pruritus**	0	no pruritus	Changes in hair/coat/skin?
	1	occasional episodes of itching	
	2	regular episodes of itching, but stops when dog is asleep	
	3	dog regularly wakes up because of itching	

Additional parameters

**Water intake**		normal	
		decreased	
		moderately increased	
		severely increased	
**Urination amount**		normal	
		decreased	
		moderately increased	
		severely increased	
**Flatulence**		normal	
		mild	
		moderate	
		severe	
**Serum cobalamin concentration (if known)**	__________ng/L on _________________________(date)		

<table>
<tr><td rowspan="2">clinical signs</td><td>When was the first time of clinical signs?</td><td>/ (mm/yy)</td></tr>
<tr><td>How long has this patient having the clinical signs?</td><td>(wk・mth・yr)</td></tr>
<tr><td colspan="2">Was this patient on any immunosuppressive drugs at the time of sample collection?</td><td>Yes / No</td></tr>
<tr><td rowspan="3">Please list all immunosuppressive drugs given up to 6 months prior to sample collection*.</td><td colspan="2">Drug name: Dose:
Regimen(i.e. q8h): Start date: End date**:</td></tr>
<tr><td colspan="2">Drug name: Dose:
Regimen(i.e. q8h): Start date: End date**:</td></tr>
<tr><td colspan="2">Drug name: Dose:
Regimen(i.e. q8h): Start date: End date**:</td></tr>
<tr><td colspan="2">Was this patient on any antibiotic drugs before/at the time of sample collection?</td><td>Yes / No</td></tr>
<tr><td rowspan="3">Please list all antibiotics given up to 6 months prior to sample collection*.</td><td colspan="2">Drug name: Dose:
Regimen(i.e. q8h): Start date: End date**:</td></tr>
<tr><td colspan="2">Drug name: Dose:
Regimen(i.e. q8h): Start date: End date**:</td></tr>
<tr><td colspan="2">Drug name: Dose:
Regimen(i.e. q8h): Start date: End date**:</td></tr>
<tr><td rowspan="2">Please tell us about cobalamin supplementation given up to 6 months prior to sample collection*.</td><td colspan="2">Drug name: Dose:
Regimen(i.e. q8h): Start date: End date**:</td></tr>
<tr><td colspan="2">Drug name: Dose:
Regimen(i.e. q8h): Start date: End date**:</td></tr>
<tr><td rowspan="3">Please list all additional medications (including supplements and nutraceuticals) given up to 6 months prior to sample collection*.</td><td colspan="2">Drug name: Dose:
Regimen(i.e. q8h): Start date: End date**:</td></tr>
<tr><td colspan="2">Drug name: Dose:
Regimen(i.e. q8h): Start date: End date**:</td></tr>
<tr><td colspan="2">Drug name: Dose:
Regimen(i.e. q8h): Start date: End date**:</td></tr>
</table>

***Please list beginning with the most recent drugs.**
****If a patient is currently on this drug, please leave "End date" blank.**
